# A service evaluation of e-triage in the osteoporosis outpatient clinic—an effective tool to improve patient access?

**DOI:** 10.1007/s11657-020-0703-1

**Published:** 2020-03-21

**Authors:** John R. Lindsay, G. Lawrenson, S. English

**Affiliations:** Osteoporosis Service, Musgrave Park Hospital, Belfast Health & Social Care Trust, Stockmans Lane, Belfast, BT9 7JB UK

**Keywords:** Osteoporosis, Triage, Electronic health care record, Patient access

## Abstract

***Summary*:**

We introduced an electronic triage system into our osteoporosis service to actively manage referral demand in a busy outpatient service. Our study demonstrated the effectiveness of e-triage in supporting alternative management pathways, through use of virtual advice and direct to investigation services, to improve patient access.

**Purpose:**

Osteoporosis referrals are increasing with awareness of the potential for prevention of fragility fracture and with complex decision making around management with long-term bisphosphonate therapy. We examined whether active triage of referrals might improve referral management processes and patient access to osteoporosis services.

**Methods:**

We implemented electronic triage (e-triage) of referrals to our osteoporosis service using the Northern Ireland electronic health care record. This included the option of ‘advice only’, direct to investigation with DXA or face-to-face appointments at the consultant-led complex osteoporosis service. We anticipated that there was scope to manage patient flow direct to investigation, or to provide referring clinicians with clinical advice without the need for a face-to-face assessment, at the consultant-led specialist service.

**Results:**

We reviewed e-triage outcomes of 809 referrals (692 F; 117 M) to osteoporosis specialist services (mean age 65 ± 16.5 years) over a 12-month period. There was a high degree of agreement for the triage category between the referring clinician and specialist services (741/809). 73.3% attended a face-to-face appointment at the consultant-led clinic, while active triage enabled direct to investigation (18.4%) or discharge (8.3%) in the remainder. The mean time between receipt of an electronic referral and e-triage was 3 days over the 12-month period as compared with 2.1 days (median 1.1 days) when annual leave periods were excluded.

**Conclusion:**

E-triage supports effective referral management in a busy osteoporosis service. Efficiency is limited by reliance on a sole clinician and 5 day working at present. There is scope to further improve systems access through multidisciplinary team working, virtual clinics and future information technology developments.

## Introduction

Post-menopausal osteoporosis is common and is associated with an increased risk of fracture [1]. Fragility fractures arising from osteoporosis affect one in two women and one in five men over the age of 50 years [2]. Fragility fractures are costly and in the UK National Health Service the hospital costs of hip fractures alone are estimated at £1.1 billion [3]. Osteoporotic fractures have significant negative impacts on quality of life with loss of independence and mobility.

In some areas within our region, demand for osteoporosis services exceeds the supply, resulting in limited access. More recently, with initiatives to support review of long-term bisphosphonate prescriptions, we have observed increasing referral demand to the specialist service from primary care.

Accessibility and long waiting times are a common problem in many publicly funded health care systems. Management of referral demand using active triaging of referrals is used in high demand specialities including neurology, given long waiting times for routine consultations [4]. Active triage can be helpful by reducing wait times, through use of alternate pathways, and to identify urgent clinical conditions, that may benefit from expedited appointment scheduling [4].

Previous electronic triage pilots for neurological conditions in our region, using an email system for new outpatient referrals, showed that most new patients and primary care clinicians found high levels of patient and clinician satisfaction [5]. In this setting over 50% were managed by email advice alone, or by email plus investigations. Almost half of the clinician’s time was saved compared with conventional consultation in this setting [5].

The Belfast Trust is the largest integrated Health and Social Care Trust in the United Kingdom, delivering care to a population of approximately 340,000 across the greater Belfast area [6]. The Osteoporosis Service at Musgrave Park Hospital provides specialist support for the Belfast Trust and wider region. Routes into the clinical service from primary care include direct access DXA within the radiology department, without the need to refer to consultant-led clinics. Nurse-led fracture liaison services are available for those aged 50–80 years, admitted with fragility fractures or recruited from fracture outpatient clinics.

Consultant-led osteoporosis clinics are available to primary and secondary care clinicians seeking specialist input for individuals with pre-existing radiologically confirmed osteoporosis, defined by DXA, using World Health Organization criteria [7]. Historically, paper based referrals were received by the clinical service, which impacted the efficiency of processing referrals.

In recent years, a secure electronic clinical communication gateway (CCG) to support electronic delivery of referrals has been introduced. The Northern Ireland Electronic Health Care Record (NIECR) provides clinicians with a comprehensive record for patient using health and social care services in Northern Ireland. The system provides clinical staff with a single view of key patient information including demographics, laboratory results, medications, allergies, diagnoses, encounters, and clinical correspondence. NIECR has evolved to include electronic triage [8].

We subsequently implemented electronic triage (e-triage) in our osteoporosis service using these systems. Options of ‘advice only’ rather than face-to-face appointments or direct to investigation with DXA have been formalised using e-triage for those deemed not to require an appointment at the consultant-led complex osteoporosis service.

## Methods

We hypothesised that active e-triage in the osteoporosis service, using the Northern Ireland Electronic Record, would support effective management of patient flow to appropriate specialist osteoporosis services.

We retrospectively evaluated e-triage outcomes for the period 1st June 2018 to 31st May 2019 by obtaining a download of the NIECR e-triage archive file. The service evaluation excluded any individuals referred by secondary care colleagues and a small number of legacy paper based referrals from primary care.

Clinical data including demographics, date and time of referral, triage and completion were reviewed. Triage priority and outcomes were examined. We anticipated that there was significant scope to manage patient flow direct to investigation or to provide referring clinicians with clinical advice, without the need for a face-to-face appointment, at the consultant-led specialist service.

Chi square testing was used to compare the proportion of patients waiting for new patient assessment before and after introduction of e-triage.

## Results

Eight hundred and nine electronic referrals were received from primary care (692 female; 117 male) over a12-month period. The age distribution was negatively skewed with a mean age of 65 ± 16.5 years (range 21–91 years).

**Fig. 1:**
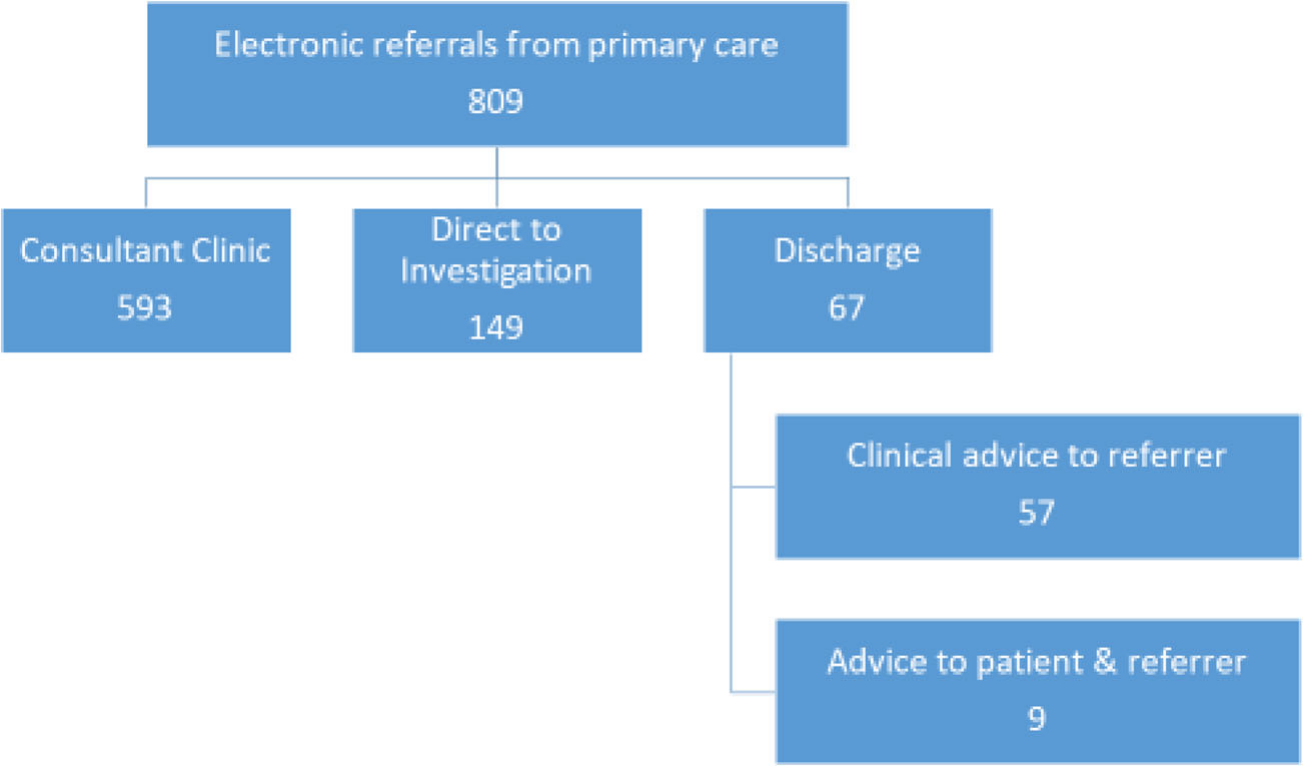
Outcomes of new patient triage process following osteoporosis specialist grading

710/809 primary care referrals were requested as routine and 99/809 as urgent. We observed high concordance in the urgency category grading between primary care and the specialist osteoporosis service (741/809). A limited number of urgent referrals (39/99) were downgraded to routine, and a small number of routine referrals (29/710) were upgraded to urgent (Fig. 1). Eighty-nine percent of the series were graded as routine following osteoporosis specialist triage.

Ninety-four of 809 referrals were aged under 50 years while 43 were aged under 40 years (female 71%/29% male). Eighty-eight percent of these patients had low bone mass within the osteoporosis or osteopenia category. Only one patient, with prior liver transplantation for autoimmune hepatitis, did not meet the entry criteria for consultant-led clinic assessment. 31/43 referrals were offered an appointment, whereas 9 were sent direct for investigation and 3 referrers were provided with virtual advice. Individuals within the age range 21–40 years presented with fragility or stress fractures (11/43), anorexia (8/43), osteogenesis imperfecta (2/43), gastrointestinal conditions (5/43), endocrine disorders (3/43), family history (2/43), inflammatory arthritis (1/43) or other relevant medical conditions pre-disposing to low bone mass.

The e-triage system permits designation of referral to the pooled osteoporosis specialist team or to a specific designated consultant. A minority of cases (*n* = 50), where assigned to a specific team member; the remainder were allocated to the next available appointment to optimise patient access.

New patient referrals were triaged to the following categories: Consultant clinic appointment (593/809), direct to investigation (149/809) or discharge (67/809) (Fig. 1). In the subgroup who were discharged (57/67) the referring primary care doctor received a letter with clinical advice and for 9/67 of the series a letter was sent to the patient as well as the referring clinician. In 61/809 cases the CCG request was redirected, as the request was seeking direct access DXA via the radiology department. In a small number of cases redirection to an alternative specialty service (*n* = 2) or to a specific consultant (*n* = 3) for advice was arranged.

**Fig. 2:**
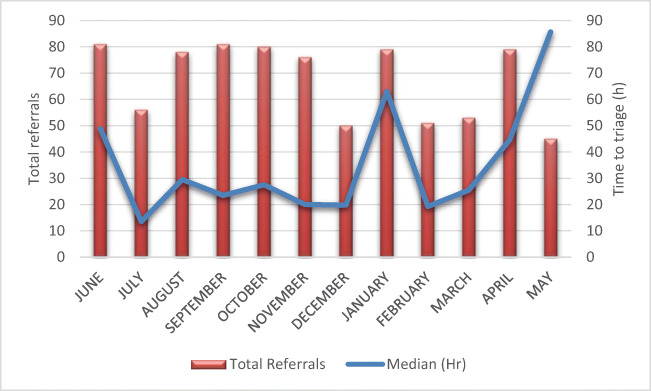
Seasonal variation in total referral activity and time to triage over 12 months

We observed seasonal variation in referral volumes across the year with troughs around July, December and May, coinciding with holiday periods within the region (Fig. 2). The mean time between receipt of an electronic referral and e-triage was 3 days over the 12-month period (Mode 1 day; median 6 days; range 20 days). When annual leave periods were excluded, during which there was no cover for e-triage, the mean time for e-triage from receipt of the referral was 2.1 days (median 1.1 days).

**Fig. 3:**
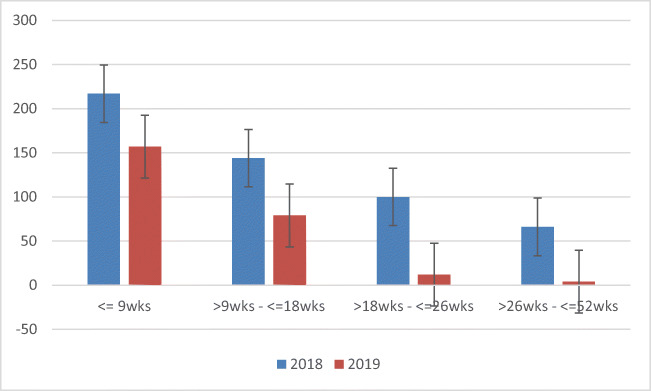
Waiting time for new patient assessment at consultant-led osteoporosis clinic

Since the inception of e-triage service in 2018 we have observed a reduction in the proportion of new patient referrals waiting for assessment <9wks, 9–18 weeks, 18–26 weeks or longer compared with the previous year (*p* < 0.0001); Fig. 3).

## Discussion

The traditional approach of direct face-to-face clinical encounters is challenging with increasing referral volumes to osteoporosis services. Electronic active triage offers an opportunity to address extensive waiting times for access to medical services through a streamlined screening process [9]. Examples of triage exist within a range of high volume referral specialties including orthopaedics, dermatology and in neurological services [4, 9, 10]. The broad purpose of triage include managing long waiting times and to ensure that individuals are directed to appropriate clinical services [11]. Triage may ensure timely access to the right patient, within the right service, at the right time [4, 9, 10]. Screening can occur at various levels across the multi-disciplinary team, and in earlier series high rates of correct allocation, according to urgency, has been achieved [10].

This service evaluation demonstrates the utility and effectiveness of e-triage in our osteoporosis service using the NIECR. We undertook e-triage of 809 referrals over a 12-month period with high concordance in the triage category across primary care and specialist services. While a majority required a face-to-face appointment at the consultant-led clinic, active triage enabled direct to investigation in over a quarter of referrals. Timely triage occurred in a most cases, although this was impacted by reliance upon a sole clinician who is responsible for the triage process. Outcomes included limiting flow to consultant clinics through use of virtual assessment using the NIECR, and provision of advice to the referring clinician or patient. During this service improvement project, we observed a 48% reduction new patient waiting lists as compared with the same timeframe in the previous year, which can be partly attributable to active triage.

Primary care specialists are in an excellent position to allocate referral urgency as main clinical care provider, however it is important to have a clear framework to guide referring clinicians. In some cases an overestimation of urgency level is noted in the literature [10]. In our series we observed that 22/710 referrals were upgraded, whereas 33/99 of primary care referrals were downgraded by the specialist team. A recent paper concerning orthopaedic triage, based on a systemic review process, showed that there was a need for standardisation of the definition of triage, the procedure of assessment and management, and measures of outcomes in orthopaedic musculoskeletal triage, to ensure best practices and outcomes for triage clinics [9]. One of the limitations in our series is that there is no clearly defined prioritisation level for individuals referring to the osteoporosis service. Strategies for allocation of clinical appointments according to imminent fracture risk across low, medium and high priority categories may be considered in the future [12].

Patterson et al. previously undertook an early process of e-mailed triage, with a prospective single cohort study in our region [5]. This series showed that triaging referrals based on a neurologist judgement appeared reasonably safe and effective [5]. Less than half of participants required a clinic appointment; 45% were managed by e-mail alone and 12% by e-mail plus investigation. GP satisfaction rates were high, measured using a Likert scale. E-mail correspondence between a GP and a neurologist was deemed to be effective, efficient and with reported excellent satisfaction rates [5]. Since then a confidential referral process from individual practices has been developed through a secure CCG system in our region, which facilitates electronic receipt of referrals. While in the present series a high proportion of referred patients did receive a face-to-face appointment, future initiatives including virtual review clinics may improve patient access further.

Our review of the literature identified limited evidence of use of e-triage or e-consult programmes in osteoporosis settings. Recently, Lee et al. reported on the geographical scope and accessibility of a centralised electronic consult programme for patients presenting with recent fractures in a veterans’ setting in North America. Low trauma osteoporotic fracture patients were evaluated from an inpatient and outpatient electronic encounter database [13]. Electronic medical records were reviewed, leading to an intervention with a bone health nurse liaison service, who coordinated and ordered the follow up of laboratory and bone density assessment, osteoporosis education and adherence follow up. In this large series 2775 fracture episodes were noted and 321 e-consults were completed. The advantage of this process enabled assessment of 53.3% of the cohort of individuals residing in the rural or highly rural areas. The nurse liaison significantly improved bisphosphonate ordering and bone mineral density testing completion rates, which were increased for both urban and rural patients. There were also substantial benefits in saving travelling time [13].

E-triage and e-consult services can be time consuming, and in one series in a neurology practice, time to review a referral averaged around 10 min but this was offset by less than half of the flagged referrals having been deemed to not require an appointment [4]. Our outcomes compare favourably to patient access arrangements within other specialty services providing osteoporosis care [14]. The mean time for our osteoporosis service e-triage from receipt of the referral was 2.1 days (median 1.1 days). This time included e-triage processing and provision of a virtual advice letter and management plan in 8.3%. We used standardised correspondence, to explain the management plan, with provision of telephone contact details for follow up to support service users. However, clearly in some cases, including in fracture liaison settings, there is still a requirement for face-to-face consults due to complex medical needs [13]. There may be a role for involvement of the multi-disciplinary input into e-triage, with input from the osteoporosis nursing team, as role substitution has been effective in other settings including orthopaedics [9].

There are several limitations within this series. Firstly, we were unable to make a direct comparison between prior paper based triage systems as this data was not prospectively collected prior to the introduction of e-triage. We did not measure the time required for provision of virtual advice, which is a limiting factor unless a clinician has protected time. In the present series, there was a reliance upon a single clinician who was providing the triage service on a 5-day basis. In addition, we have not had an opportunity to follow up individuals who have received virtual advice in terms of bone health or other clinical outcomes nor was there an assessment of bisphosphonate prescription use or medication concordance. We did not review satisfaction rates of service users, following e-triage. However, to our knowledge no adverse feedback from service users was received. Future ICT initiatives within our region, including the encompass programme, may offer further opportunities to support transformed digital integrated care across Northern Ireland in the coming years [15].

In summary, this service evaluation highlights the utility of e-triage systems to manage referral flow into a busy osteoporosis service. Active e-triage facilitates timely clinical advice to referrers or the option of direct to investigation. Our current e-triage system is limited by reliance on a sole clinician and 5 day working at present. However, there is potential further scope to improve processes through multidisciplinary team working, implementation of virtual clinics and future information technology solutions.

## References

[1] Eastell R, Rosen CJ, Black DM, Cheung AM, Murad MH, Shoback D (2019). Pharmacological management of osteoporosis in postmenopausal women: an Endocrine Society clinical practice guideline. J Clin Endocrinol Metab.

[2] Van Staa TP, Dennison EM, Leufkens HGM, Cooper C (2001). Epidemiology of fractures in England and Wales. Bone.

[3] Effective Secondary Prevention of Fragility Fractures: Clinical Standards for Fracture Liasison Services. Royal Osteoporosis Society (2019). Available at: https://theros.org.uk/media/100702/royal-osteoporosis-society-clinical-standards-for-fracture-liaison-services.pdf. Accessed 4th Oct 2019

[4] Brilla R, Gardon S, Jantzen A, Weiss A (2018). Referral management: which patients are deemed not appropriate for neurologic consultation, and what happens to them?. Clin Neurol Neurosurg.

[5] Patterson V, Humphreys J, Chua R (2004). Email triage of new neurological outpatient referrals from general practice. J Neurol Neurosurg Psychiatry.

[6] Belfast Health & Social Care Trust Annual Report 2017-18. Available at http://www.belfasttrust.hscni.net/pdf/BHSCT%20Annual%20Report%20and%20Accounts%202017-18.pdf. Accessed 4th Oct 2019

[7] World Health Organization (1994). Assessment of fracture risk and its application to screening for postmenopausal osteoporosis: technical report series 843.

[8] Northern Ireland Electronic Healthcare record (NIECR). Available at: https://www.nidirect.gov.uk/articles/northern-ireland-electronic-care-record-niecr. Accessed 4th Oct 2019

[9] Morris JH, James RE, Davey R, Waddington G (2015). What is orthopaedic triage? A systematic review. J Eval Clin Pract.

[10] Deluca J, Goldschmidt A, Eisendle KJ (2016). Analysis of effectiveness and safety of a three-part triage system for the access to dermatology specialist health care. Eur Acad Dermatol Venereol.

[11] Bennett K, de Boisanger L, Moreton F, Davenport R, Stone J (2019). The safety of using active triage to provide advice rather than a face-to-face neurology outpatient appointment. J R Coll Physicians Edinb.

[12] Khosla S (2019). Personalising osteoporosis treatment for patients at high risk of fracture. Lancet Diabetes Endocrinol.

[13] Lee RH, Lyles KW, Pearson M, Barnard K, Colón-Emeric C (2014). Osteoporosis screening and treatment among veterans with recent fracture after implementation of an electronic consult service. Calcif Tissue Int.

[14] Widdifield J, Tu K, Carter Thorne J, Bombardier C, Michael Paterson J, Liisa Jaakkimainen R, Wing L, Butt DA, Ivers N, Hofstetter C, Lyddiatt A, Ahluwalia V, Bernatsky S (2017). Patterns of care among patients referred to rheumatologists in Ontario, Canada. Arthritis Care Res (Hoboken).

[15] Health and Social Care Board encompass programme. http://www.hscboard.hscni.net/encompass/. Accessed 23rd Dec 2019

